# Extent of tumor fibrosis/hyalinization and infarction following neoadjuvant radiation therapy is associated with improved survival in patients with soft‐tissue sarcoma

**DOI:** 10.1002/cam4.4428

**Published:** 2021-11-27

**Authors:** Sneha R. Rao, Alexander L. Lazarides, Bruce L. Leckey, Whitney O. Lane, Julia D. Visgauss, Jason A. Somarelli, David G. Kirsch, Nicole A. Larrier, Brian E. Brigman, Dan G. Blazer, Diana M. Cardona, William C. Eward

**Affiliations:** ^1^ Department of Orthopaedics Duke University Durham North Carolina USA; ^2^ Department of Pathology Duke University Durham North Carolina USA; ^3^ Department of Surgery Duke University Durham North Carolina USA; ^4^ Department of Medicine Duke University Durham North Carolina USA; ^5^ Department of Radiation Oncology Duke University Durham North Carolina USA

**Keywords:** fibrosis, neoadjuvant radiation therapy, sarcoma, soft tissue

## Abstract

**Introduction:**

Current standard of care for most intermediate and high‐grade soft‐tissue sarcomas (STS) includes limb‐preserving surgical resection with either neoadjuvant radiation therapy (NRT) or adjuvant radiation therapy. To date, there have been a few studies that attempt to correlate histopathologic response to NRT with oncologic outcomes in patients with STS.

**Methods:**

Using our institutional database, we identified 58 patients who received NRT followed by surgical resection for primary intermediate or high‐grade STS and 34 patients who received surgical resection without NRT but did receive adjuvant radiation therapy or did not receive any radiation therapy. We analyzed four histologic parameters of response to therapy: residual viable tumor, fibrosis/hyalinization, necrosis, and infarction (each ratiometrically determined). Data were stratified into two binary groups. Unadjusted, 5‐ and 10‐year overall survival, and relapsed‐free survival (RFS) were calculated using the Kaplan–Meier method.

**Results:**

Analysis of pathologic characteristics showed that patients treated with NRT demonstrate significantly higher tumor infarction, higher tumor fibrosis/hyalinization, and a lower percent viable tumor compared with patients not treated with NRT (*p* < 0.0001). Based on Kaplan–Meier curve analysis and multivariate cox proportional hazard model for OS and RFS, patients treated with NRT and showing >12.5% tumor fibrosis/hyalinization have significantly higher overall survival and recurrence‐free survival at 5 and 10 years.

**Discussion and Conclusion:**

We have identified three histopathologic characteristics—fibrosis, hyalinization, and infarction—that may serve as predictive biomarkers of response to NRT for STS patients. Future prospective studies will be needed to confirm this association.

## Graphical abstract

For patients treated with soft‐tissue sarcoma and neoadjuvant therapy, we have identified pathologic markers found at the time of surgical resection which are unique to tumors treated with NRT. Among these markers, higher percent fibrosis/hyalinization and lower percent tumor infarction were associated with improved overall survival and disease‐free survival.

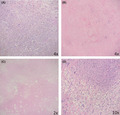

## 1 INTRODUCTION

Current standard of care for intermediate to high‐grade soft‐tissue sarcoma (STS) includes neoadjuvant radiation therapy (NRT) followed by limb‐preserving surgical resection.^1^, ^2^ The landmark clinical trial by O’Sullivan et al. comparing NRT to adjuvant radiation therapy showed essentially no difference in oncologic outcomes between the two groups, but benefits in late effects from NRT were a result of utilizing a lower total radiation dose and radiation field size.^3^ Indeed, NRT and the benefits it confers facilitate limb salvage surgeries that provide adequate tumor resection while maximizing postoperative functional outcomes.^2^ Despite the benefits of NRT, local and distant treatment failures do occur, and new prognostic markers are needed that help predict meaningful oncologic outcomes for patients who receive NRT and surgical resection.^3^, ^4^, ^5^

Histopathologic analysis at the time of tumor resection following NRT has often been limited to the measurement of tumor necrosis and treatment effect. The data investigating the utility of percent tumor necrosis in STS treated with NRT shows conflicting evidence. Canter et al. and Shah et al both utilized the same tumor necrosis threshold for complete pathologic response, >95%, and did not identify a statistically significant difference in survival outcomes.^6^, ^7^, ^8^ Data from the European Organization for Research and Treatment of Cancer‐Soft‐Tissue and Bone Sarcoma Group have looked beyond percent tumor necrosis and quantified percent residual viable cells, necrosis, hyalinization/fibrosis, and infarction in surgically resected STS treated with NRT.^9^ Among the patients in this cohort, hyalinization/fibrosis was found to correlate with improved survival.^9^

Given the findings of the European Sarcoma cohort, we aimed to identify histopathologic characteristics unique to patients who received NRT and surgical resection compared with patients who received surgical resection without NRT. In addition, we aimed to correlate histopathologic characteristics of the resected tumor with 5‐ and 10‐year overall survival (OS) and recurrence‐free survival (RFS) in patients with STS of the trunk and extremities. We hypothesized that patients undergoing NRT followed by surgical resection will have a unique histopathologic profile that is associated with improved OS and RFS.

## 2 METHODS

Patients over the age of 18 undergoing surgical resection for intermediate or high‐grade STS of the limbs and trunk between 1998 and 2015 at a single institution were identified retrospectively using the DEDUCE. We identified 58 patients who received NRT followed by surgical resection for primary intermediate or high‐grade STS and 34 patients with intermediate to high‐grade STS who received surgical resection with or without adjuvant RT. Within the group that did not receive NRT, 22 patients received adjuvant radiation therapy; however, the remainder of patients did not receive adjuvant RT due to the clinical impression that wide surgical resection was adequate to achieve local control or other patient clinical factors such as progressive disease. Patients who received preoperative chemotherapy were excluded from the study. Neoadjuvant radiation therapy regimens were determined by the primary radiation oncologist for each patient.

Patient age was determined from the date of surgical resection. Tumor location was classified as upper extremity (distal to shoulder and axilla), lower extremity (distal to groin), and trunk. STS presenting in the thorax, abdomen, retroperitoneum, or pelvis were not included in this study. Tumor grade was obtained from the histopathologic report at the time of surgical resection. Tumor size was classified as <5 cm, between 5 cm and 10 cm, and >10 cm, the largest dimension of each tumor was analyzed. Margins were classified as negative if the margin was free of tumor or positive if the tumor extended to margin. The date of local tumor recurrence was established from either the first imaging finding, physical exam, or biopsy of the tumor bed. The date of distant metastases was established from the imaging or biopsy findings of metastatic lesions.

**FIGURE 1:**
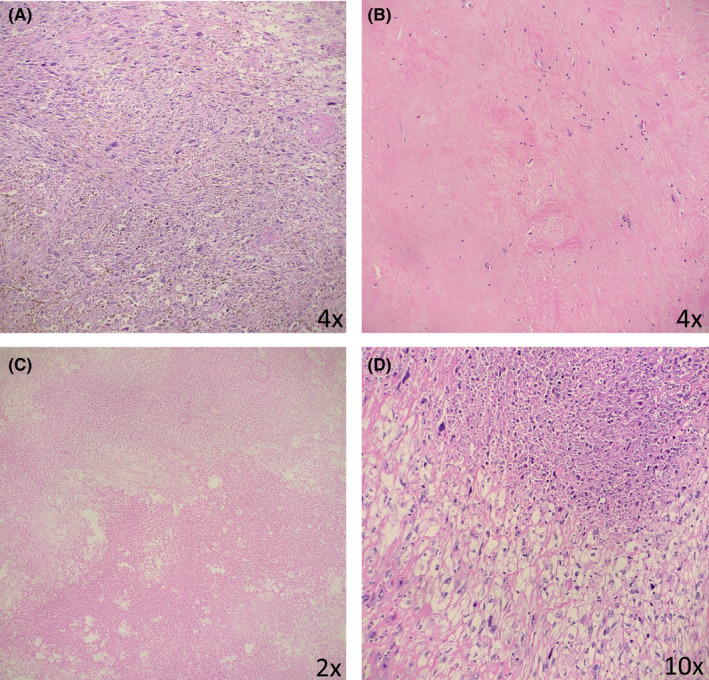
Representative images of histopathologic characteristics for remaining viable tumor (A), fibrosis/hyalinization (B), infarction (C), and tumor necrosis (D)

Semi‐quantitative analysis of resected soft‐tissue tumors of the extremity and trunk was done concurrently by two pathologists (B.L. and D.C.) evaluating four histologic parameters of response to therapy in the tumor bed (as a percentage): residual viable tumor (tumor cells with intact nuclear details), fibrosis/hyalinization (dense amorphous eosinophilic material or collagen deposition), necrosis (ghost tumor cells with associated nuclear/cellular debris and neutrophilic inflammation), and infarction (nonviable tumor lacking an inflammatory response or nuclear/cellular debris). The histopathologic images of the four parameters are shown in Figure 1. The histopathologic parameters were collected by retrospective analysis of archived pathology slides taken at the time of surgical resection of the tumor. On average, one section of tumor/cm of tumor mass was submitted per the institutional protocol for each tumor and evaluation of these categories was performed on each section and averaged for the final overall percentages in each case. The pathologists divided the cases such that each case was reviewed by one pathologist. The number of slides analyzed per patient ranged from 5 slides to 30 slides. The pathologists performing histopathologic analysis were not blinded to patient demographic information; however, they were blinded to oncologic survival outcomes.

To determine the cutoff point to stratify histopathologic data for statistical analysis, ROC curves were plotted to identify the point of maximum correlation with survival. To establish the value that most correlated with mortality in this data set, death within 5 years as a categorical “Yes” or “No” variable was plotted against the continuous variable of interest (i.e., percent infarction or amount of coagulative necrosis). Then, receiver operative characteristic (ROC) curves were generated by plotting sensitivity (*Y*‐axis) vs. 1 − specificity (*X*‐axis) (Figure S1). From these ROC curves, the value for each continuous variable that resulted in maximum the area under the curve and achieved the best‐combined sensitivity and specificity was identified. Based on the ROC curve analysis, data were stratified into two groups, greater than or less than 27% of percent tumor infarction, 12.5% of fibrosis/hyalinization, presence or absence of coagulative necrosis, and greater than or less than 5% viable tumor. Overall survival and recurrence‐free survival were calculated from the date of surgical resection to the current date and date of disease relapse (local or metastatic), respectively. Unadjusted 5‐ and 10‐year overall survival and 5‐ and 10‐year recurrence‐free survival was calculated using the Kaplan–Meier method and differences between groups were analyzed using the log‐rank test with *p* < 0.05. Univariate analysis of patient demographics and histopathologic characteristics with oncologic outcomes were performed with Chi‐squared analysis for categorical variables and Student's *t* test for continuous variables. Multivariate analysis of histopathologic characteristics with oncologic outcomes was performed using the Cox proportional hazard model. Statistical analysis was performed using JMP version 13.1 SAS Institute Inc., Cary, NC.

## 3 RESULTS

### 3.1 Demographics and Oncologic outcomes

Patient demographic data and oncologic outcomes for the 58 patients treated with neoadjuvant radiation therapy and 34 patients treated with surgical resection without neoadjuvant radiation therapy are shown in Table 1. For patients in the NRT treatment group, the average age of the patients was 64 years; 50% of patients were female, and the majority of patients identified as Caucasian (Table 1). The most common STS tumor subtype within this cohort was undifferentiated pleomorphic sarcoma. Tumors in the “other” category included: malignant fibrous histiocytoma, myxofibrosarcoma, malignant peripheral nerve sheath tumor (MPNST), synovial sarcoma, epithelioid sarcoma, and alveolar soft part sarcoma (Table 1). The most common presentation of STS was in the lower extremity (69%) and a majority of tumors were high grade (70%). The average size of tumors was 9.1 cm in the largest dimension (Table 1). The majority of STS treated with NRT had margin negative surgical resections; four patients had positive margins. The median overall survival for patients in the NRT cohort was 6.6 years with 41 (60%) patients surviving to 5 years (Table 1). In this cohort, 31 patients (53%) had either metastasis (24 patients), local recurrence (two patients), or both (five patients) and the median time to recurrence was 1.2 years with an average follow‐up of 4.7 years (ranging from 6 months to 12.2 years) and median follow‐up time of 3 years (Table 1). Univariate analysis of the association of patient demographics such as age, gender, race, and tumor location shows no statistically significant correlation with 5‐ and 10‐year recurrence‐free survival (RFS) and overall survival (OS) (Table 2).

**TABLE 1:** Patient cohort demographics and oncologic outcomes for patients treated with and without NRT

Patient factors	Patients treated with NRT, No. (%)	Patients treated without NRT, No. (%)	*p*‐value
Mean age (y)	64	56	0.04
Gender			0.27
Male	29 (50%)	21(62%)	
Female	29 (50%)	13 (38%)	
Race			0.21
African‐American	17 (29%)	11 (32%)	
Caucasian	37 (64%)	21 (62%)	
Other	4 (7%)	2 (6%)	
Tumor type			0.3
Undifferentiated	21 (37%)	13 (38%)	
Pleomorphic sarcoma			
Leiomyosarcoma	9 (15%)	6 (18%)	
Liposarcoma	9 (15%)	1 (3%)	
Other	19 (33%)	14 (41%)	
Tumor location			0.06
Lower extremity	39 (69%)	18 (53%)	
Upper extremity	13 (23%)	10 (29%)	
Trunk	2 (4%)	6 (18%)	
Groin	2 (4%)		
Tumor size	9.1 cm	8.8 cm	0.9
Tumor grade			0.43
Grade 2	17 (30%)	8 (28%)	
Grade 3	40 (70%)	21(72%)	
Margins			0.051
Negative	53 (93%)	27 (79%)	
Positive	4 (7%)	7 (21%)	
Median overall survival	6.6 years	9.1 years	0.75
Median time to recurrence (LR or DM)	1.2 years	1.6 years	0.87

Abbreviations: LR, local recurrence; DM, distant metastasis.

**TABLE 2:** Univariate analysis of patient demographics and 5‐year and 10‐year recurrence‐free survival (RFS) and overall survival (OS) in the NRT cohort

Parameters	*n*	5‐year RFS (%)	*p*‐value	10‐year RFS (%)	*p*‐value	5‐year OS (%)	*p*‐value	10‐year OS (%)	*p*‐value
Age			0.63		0.58		0.67		0.76
<53	14	50		33.3		64.3		56.3	
53–65	18	38.5		38.5		53.9		43.1	
65–75	13	57.8		57.7		76.2		63.5	
>75	13	55.6		55.6		66.7		55.6	
Sex			0.59		0.62		0.23		0.15
Male	29	47.5		43.8		72.4		65.2	
Female	29	54.5		50.4		57.9		45.4	
Race			0.21		0.29		0.29		0.43
Caucasian	37	59.2		53.3		72.6		60	
African‐American	17	39.2		39.2		52.9		46.3	
Other	4	25		25		50		50	
Site			0.81		0.83		0.81		0.55
UE	13	53.9		53.9		69.2		69.2	
LE	39	53.2		47.6		62.9		49.7	
Other	4	25		25		75		75	

Note

A *p* value of <0.05 was considered statistically significant.

For patients that were treated with surgical resection *without* neoadjuvant radiation therapy, the average age of the patients was 56 years, 21 (62%) patients were male, and the majority of patients identified as Caucasian (Table 1). The most common tumor subtype was undifferentiated pleomorphic sarcoma and other category which included fibrous histiocytoma, dermatofibrosarcoma protuberans (DFSP), malignant peripheral nerve sheath tumor (MPNST), myxoid chondrosarcoma, synovial sarcoma, and myxofibrosarcoma (Table 1). The majority of tumors were localized to the lower extremity and a majority of tumors were high grade (Table 1). The average tumor size in the largest dimension was 8.8 cm with 9 tumors measuring <5 cm, 11 tumors between 5 and 10 cm, and 9 tumors measuring larger than 10 cm (Table 1). This cohort of patients had a larger percentage of tumors with positive margins at resection with 7 tumors (21%); 34 tumor resections had negative margins (79%) (Table 1). The median overall survival for this cohort that did not receive NRT was 9.1 years with 24 patients (71%) surviving to 5 years (Table 1). In this cohort, 16 patients (47%) had either metastatic disease (five patients), local recurrence (six patients), or both local recurrence and metastatic disease (five patients), and the median time to recurrence was 1.6 years (Table 1). The average follow‐up time in this cohort was 3.1 years with a median follow‐up of 1.3 years, there was no statistical difference in follow‐up time between patients who received NRT and patients treated without NRT based on the Student's *t* test with the p‐value of 0.2. Univariate analysis comparing patient demographics between the patients who received NRT and patients treated without NRT revealed a significantly higher age in the NRT treated group (Table 1).

### 3.2 Histopathologic characteristics

**FIGURE 2:**
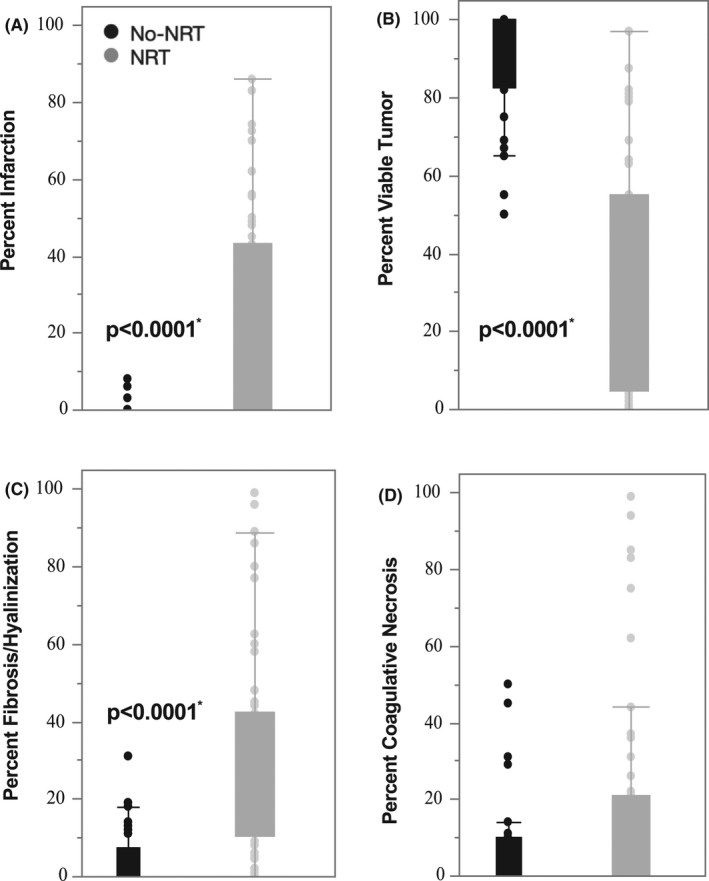
Patients treated with NRT demonstrate significantly higher tumor infarction, higher tumor fibrosis/hyalinization, and a lower percent viable tumor compared with patients not treated with NRT. The percent coagulative necrosis was not significantly different (*p* = 0.045). Comparison via Student's *t* test analysis

A summary of the four histopathologic characteristics identified in this study: infarction, viable tumor remaining, fibrosis/hyalinization, and coagulative necrosis is detailed in Figure 2 for both cohort groups. Among the histopathologic characteristics, STS tumors that were treated with NRT had significantly lower viable tumor remaining, higher percent infarction, and higher percent fibrosis/hyalinization compared with STS tumors that were surgically resected with no NRT, with the *p*‐value of <0.0001. Interestingly, the percent of coagulative necrosis did not differ between the two cohorts.

**FIGURE 3:**
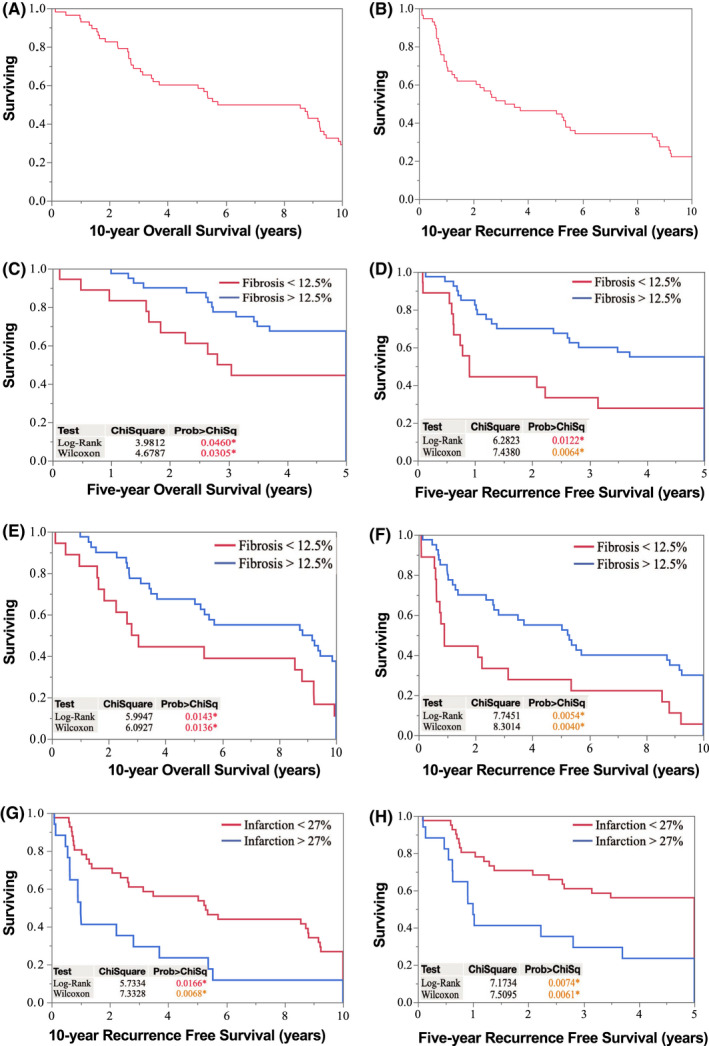
Kaplan–Meier curve analysis for patients treated with NRT. Overall 10‐year survival and 10‐year recurrence‐free survival of patients treated with NRT and surgical resection for STS. (A and B) Patients with fibrosis/hyalinization >12.5% at the time of surgical resection have significantly higher 5‐year overall survival, higher 10‐year overall survival, higher 5‐year recurrence‐free survival, and higher 10‐year recurrence‐free survival. (C‐F) Patients with lower percent tumor infarction at the time of surgical resection have significantly higher 5‐ and 10‐year recurrence‐free survival. (G and H) Kaplan–Meier curve analysis shown for panels (A–G), *p* value of <0.05 was considered statistically significant

Kaplan–Meier analysis of the three histopathologic characteristics unique to tumors treated with NRT demonstrates that tumors with >12.5% of fibrosis/hyalinization correlated significantly with higher 5‐ and 10‐year overall survival and with 5‐ and 10‐year recurrence‐free survival with the *p*‐value of <0.05 (Figure 3). Although tumor infarction was greater following NRT, an amount of tumor infarction lower than 27% was found to be associated with significantly higher 5‐ and 10‐year recurrence‐free survival. As shown in Table 3, univariate analysis of tumor characteristics, such as tumor size, histologic subtype, grade, and resection margin in addition to histopathologic characteristics, identified tumor fibrosis/hyalinization and tumor infarction as predictive of oncologic outcomes (Table 3). For patients with >12.5% tumor fibrosis/hyalinization, 5‐ and 10‐year disease‐free survival was 59.7% and 56.7%, respectively, compared with disease‐free survival of 31.8% and 25.4% in patients with <12.5% tumor fibrosis/hyalinization (Figure 3). For patients with tumor infarction <27%, 5‐ and 10‐year recurrence‐free survival was 60.3% and 54.6%, respectively, compared with recurrence‐free survival of 29.4% in patients with tumor infarction >27% (Figure 3). Unlike fibrosis/hyalinization, tumor infarction did not correlate with 5‐ and 10‐year overall survival in the Kaplan–Meier survival analysis. Analysis of percent of residual viable tumor and presence or absence of coagulative necrosis showed no statistical difference in improved overall survival or recurrence‐free survival.

**TABLE 3:** Univariate analysis of tumor characteristics and histopathologic tumor characteristics with 5‐year and 10‐year recurrence‐free survival (RFS) and overall survival (OS) in the NRT cohort

Parameter	*n*	5‐year RFS (%)	*p*‐value	10‐year RFS (%)	*p*‐value	5‐year OS (%)	*p*‐value	10‐year OS (%)	*p*‐value
Tumor size			0.12		0.19		0.16		0.06
<5	11	72.7		62.3		81.8		81.8	
>5	47	46.1		43.6		61.3		42.1	
Histologic subtype			0.48		0.41		0.45		0.58
UPS	15	33.3		0.07		53.3		52.5	
Liposarcoma	9	55.6		22.2		66.7		22.2	
Leiomyosarcoma	9	22.2		22.2		33.3		33.3	
Other	19	60		32		72		13	
Grade			0.19		0.15		0.09		0.62
Intermediate	17	64.2		64.2		81.9		57.5	
High	40	44.5		41.6		57.5		54.9	
Margins			0.22		0.26		0.55		0.76
Negative	53	53.1		48.7		65.7		55.5	
Positive	4	25		25		50		50	
Treatment Effect			0.88		0.88		0.2		0.095
High	10					50		25	
Intermediate	2					0		0	
Minimal	5					80		80	
Residual viable tumor			0.97		0.78		0.9		0.78
<5	15	53.3		53.3		66.7		59.3	
>5	43	50.3		44.7		64.7		53.7	
Fibrosis/hyalinization			0.015		0.009		0.013		0.03
>12.5%	40	59.7		56.7		74.6		62.7	
<12.5%	18	31.8		25.4		44.4		38.9	
Coagulative necrosis			0.81		0.95		0.1		0.08
Present	19	52.6		52.6		52.6		40.1	
Absent	39	50.8		44.5		71.3		62.5	
Infarction			0.009		0.017		0.054		0.068
>27%	41	29.4		29.4		47.1		40.3	
<27%	17	60.3		54.6		72.9		61.6	

Note

*p* values of <0.05 were considered statistically significant.

**FIGURE 4:**
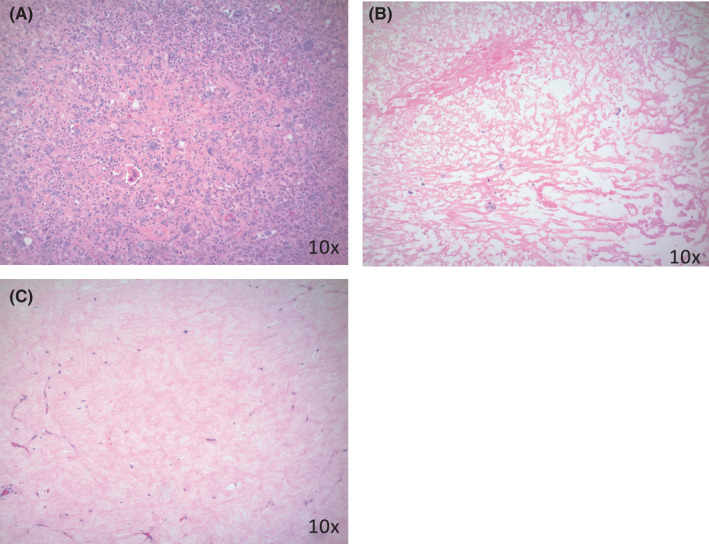
Representative histopathologic images of tumors with low percent infarction and fibrosis/hyalinization (A), high percent infarction (B), and high percent fibrosis/hyalinization

We conducted a multivariate analysis to identify which histopathologic characteristics were independently associated with overall and recurrence‐free survival; we further stratified with and without the inclusion of tumor grade to control for the independent contribution of tumor grade (Tables 4 and 5). Tumors with >12.5% fibrosis/hyalinization were found to be independently associated with significantly higher 5‐ and 10‐year recurrence‐free survival. Percent tumor infarction >27% was independently associated with increased risk of death for 5‐ and 10‐year recurrence‐free survival and 5‐ and 10‐year overall survival. These trends were observed whether tumor grade was included (Table 5) or excluded (Table 4) from the multivariate analysis. Representative histopathologic images of tumors with high and low tumor infarction and fibrosis/hyalinization are shown in Figure 4. Other histopathologic characteristics, percent viable tumor, coagulative necrosis, and tumor grade were not found to be independently correlated with disease‐free or overall survival.

**TABLE 4:** Multivariate analysis of histopathologic tumor characteristics excluding tumor grade after neoadjuvant radiation therapy with 5‐year and 10‐year recurrence‐free survival and overall survival

Variable	Reference	Risk ratio	*p*‐value	Lower 95%	Upper 95%
5‐year survival					
Viable tumor > 5%	Viable tumor < 5%	1.24	0.69	0.43	3.53
Fibrosis > 12.5%	Fibrosis < 12.5%	0.41	0.06	0.17	1.03
Coagulative necrosis	No coagulative necrosis	2.20	0.11	0.84	5.78
Infarction > 27%	Infarction < 27%	2.99	0.02	1.16	7.69
10‐year survival					
Viable tumor > 5%	Viable tumor < 5%	1.24	0.65	0.48	3.18
Fibrosis > 12.5%	Fibrosis < 12.5%	0.50	0.09	0.22	1.13
Coagulative necrosis	No coagulative necrosis	2.14	0.08	0.91	5.04
Infarction > 27%	Infarction < 27%	2.60	0.03	1.11	6.11
5‐year recurrence‐free survival					
Viable tumor > 5%	Viable tumor < 5%	0.98	0.96	0.40	2.37
Fibrosis > 12.5%	Fibrosis < 12.5%	0.39	0.02	0.18	0.87
Coagulative necrosis	No coagulative necrosis	1.12	0.80	0.49	2.56
Infarction > 27%	Infarction < 27%	2.79	0.01	1.28	6.07
10‐year recurrence‐free survival					
Viable tumor > 5%	Viable tumor < 5%	1.05	0.92	0.44	2.51
Fibrosis > 12.5%	Fibrosis < 12.5%	0.37	0.01	0.17	0.80
Coagulative necrosis	No coagulative necrosis	0.96	0.93	0.43	2.18
Infarction > 27%	Infarction < 27%	2.53	0.02	1.18	5.40

Note

The relative risk of >1 indicate an increased risk of death associated with the variable compared with the reference.

**TABLE 5:** Multivariate analysis of histopathologic tumor characteristics including tumor grade after neoadjuvant radiation therapy with 5‐year and 10‐year recurrence‐free survival and overall survival

Variable	Reference	Risk ratio	*p*‐value	Lower 95%	Upper 95%
5‐year survival					
Viable tumor > 5%	Viable tumor < 5%	1.33	0.59	0.46	3.80
Fibrosis > 12.5%	Fibrosis < 12.5%	0.43	0.08	0.17	1.10
Coagulative necrosis	No coagulative necrosis	1.94	0.18	0.73	5.15
Infarction > 27%	Infarction < 27%	2.73	0.04	1.06	7.00
Grade 3	Grade 2	1.92	0.31	0.54	6.79
10‐year survival					
Viable tumor > 5%	Viable tumor < 5%	1.24	0.66	0.48	3.21
Fibrosis > 12.5%	Fibrosis < 12.5%	0.54	0.17	0.23	1.30
Coagulative necrosis	No coagulative necrosis	2.31	0.06	0.95	5.58
Infarction > 27%	Infarction < 27%	2.72	0.03	1.13	6.54
Grade 3	Grade 2	1.03	0.95	0.39	2.74
5‐year recurrence‐free survival					
Viable tumor > 5%	Viable tumor < 5%	1.00	0.99	0.41	2.43
Fibrosis > 12.5%	Fibrosis < 12.5%	0.38	0.02	0.16	0.87
Coagulative necrosis	No coagulative necrosis	1.01	0.98	0.43	2.35
Infarction > 27%	Infarction < 27%	2.56	0.02	1.17	5.61
Grade 3	Grade 2	1.22	0.68	0.47	3.21
10‐year recurrence‐free survival					
Viable tumor > 5%	Viable tumor < 5%	1.07	0.89	0.44	2.57
Fibrosis > 12.5%	Fibrosis < 12.5%	0.40	0.03	0.18	0.92
Coagulative necrosis	No coagulative necrosis	0.95	0.90	0.41	2.17
Infarction > 27%	Infarction < 27%	2.42	0.02	1.12	5.23
Grade 3	Grade 2	1.35	0.53	0.52	3.52

Note

The relative risk of >1 indicate an increased risk of death associated with the variable compared with the reference.

## 4 DISCUSSION

In this retrospective review of STS treated with combination NRT and surgical resection and STS treated with surgical resection without NRT, we identified three histopathologic characteristics that reliably distinguish STS treated with NRT from STS treated without NRT: percent viable tumor, tumor infarction, and fibrosis/hyalinization. Of these three histopathologic markers, specific parameters were identified which are associated with better outcomes. Tumors with >12.5% fibrosis/hyalinization or tumors with <27% tumor infarction had statistically higher overall survival and higher recurrence‐free survival both in Kaplan–Meier curve analysis and in multivariate analysis. These results provide further evidence that fibrosis/hyalinization and tumor infarction are predictive of overall survival and recurrence‐free survival independent of tumor grade in a cohort of STS patients treated with NRT.

Prior studies searching for prognostic histopathologic markers for STS treated with NRT have often focused on tumor necrosis. This rationale extrapolates from the known prognostic role of tumor necrosis in patients with osteosarcoma and Ewing sarcoma treated with preoperative chemotherapy. For these bone sarcomas, tumor necrosis after preoperative chemotherapy is a prognostic marker for oncologic outcomes.^10^, ^11^ The results of these studies in bone sarcomas have led to percent tumor necrosis becoming a standardized pathologic measurement for STS at the time of surgical resection even though necrosis has not been consistently associated with oncologic outcomes in patients with other sarcomas. For patients with STS, tumor percent necrosis following neoadjuvant therapy does not appear to be a reliable prognostic indicator for oncologic outcomes, both for patients treated with NRT and for those treated with chemotherapy.^8^ Mullen et al. studied a similar cohort of 113 patients with intermediate and high‐grade STS who received neoadjuvant chemoradiotherapy and found that tumor necrosis at the time of surgical resection did not correlate with oncologic outcomes.^12^ Shurell‐Linehan et al described favorable outcomes in patients with high‐risk malignant peripheral nerve sheath tumors (MPNST) receiving neoadjuvant therapy and having a pathologic response to therapy of >90% necrosis. However, this included neoadjuvant chemotherapy and radiation therapy. This type of response has not been described in other subtypes of STS.^13^ In the present study, the percent necrosis following NRT was not found to correlate with any oncologic outcomes, suggesting that necrosis alone after NRT does not contribute to prognosis in STS patients.

Our study addresses this deficit by seeking to identify other relevant histopathologic hallmarks of treatment effect that (1) are unique to tumors treated with NRT and (2) are correlated with oncologic outcomes. In this study, there was no significant difference between the percent necrosis in tumors treated with NRT and tumors treated without NRT. This could be explained by the fact that tumor necrosis varies widely between different tumors rather than being solely an indicator of NRT treatment efficacy.^8^, ^14^ For example, Gannon et al demonstrated that tumor necrosis in STS patients treated with neoadjuvant therapy was associated with poorer overall survival, distant metastasis‐free survival, and progression‐free survival.^15^ However, in this study, necrosis was highly correlated with size and grade, two factors which are independently associated with poor outcomes.^15^ Indeed, in both the French Federation of Cancer Centers Sarcoma Group and the NCI tumor grading scheme, quantitative tumor necrosis is a component of assigning tumor grade.^16^ Thus, tumor necrosis is a confounding variable in predicting oncologic outcomes for patients with NRT^14^, ^16^ as it is a variable feature of these tumors rather than a reliable marker of treatment effect.

Whereas percent necrosis was not predictive of outcome in our patient cohort, we have identified three specific histopathologic markers that differentiate tumors treated with NRT and those treated without any preoperative therapies independent of tumor grade. The results of this study along with the European sarcoma data from Schaefer et al both showed that tumor fibrosis/hyalinization correlates with favorable oncologic outcomes, and percent viable tumor was not found to correlate with outcomes in either study.^9^ In the cohort of patients reported in the current study, tumors with >12.5% fibrosis/hyalinization had statistically higher overall survival and recurrence‐free survival at both 5‐ and 10‐year time points. Similarly, Schaefer et al demonstrated that higher levels of tumor fibrosis/hyalinization correlated with improved overall survival and 5‐year recurrence‐free survival.^9^ Hyalinization is the pathologic process by which a normal tissue transition into a unique, homogeneous material. This term first came into usage in the early twentieth century and derives from the fact that such material appeared glassy and pink after staining with hematoxylin and eosin (the Greek word *hyalinos* means glass or crystal). Hyalinization is a feature of a number of specific tumors and pathologic processes. In STS, it is generally seen only as a result of prior treatment and, therefore, it is less likely to be confounded by tumor grade and size, as has been shown to occur with tumor necrosis.

In addition to identifying fibrosis/hyalinization as a potential novel prognostic measure, lower percent tumor infarction was also identified as a favorable prognostic indicator for recurrence‐free survival at both 5‐ and 10‐year time points using both the Kaplan–Meir curve analysis and Cox proportional hazards model. This result is consistent with the findings of at least one other group. In the European data, lower percent tumor infarction was also found to be a favorable prognostic indicator for 5‐year overall survival (9). The European cohort had a relatively high proportion of myxofibrosarcoma patients (25%) and outcomes were limited to 5‐year overall survival and recurrence‐free survival, whereas our study incorporates longer‐term outcomes and a broader variety of histologic subtypes. Our study categorized fibrosis/hyalinization and percent tumor infarction using ROC analysis to identify points of maximum correlation as greater than or less than 12.5% and 27%, respectively. Schaefer et al defined the histopathologic markers as four quartiles rather than binary groups. While both studies arrived at the same conclusion that higher percent tumor fibrosis/hyalinization and lower percent tumor infarction are favorable prognostic factors, the biologic basis for these outcomes remains unclear and warrants further investigation. The data provided in this cohort, in combination with the European cohort, provide compelling evidence to further investigate the three histopathologic markers associated with treatment effect in STS treated with NRT.^9^ We suggest that fibrosis/ hyalinization and infarction should be included in the pathologic description of all resected STS treated with NRT.

One limitation of this study is that it uses retrospective observations in an attempt to identify prognostic indicators. Since radiation therapy treatment regimens were not controlled and though the majority of patients received NRT at the same institution, this was not universally the case. Because we are a tertiary referral center for sarcoma patients in our region, some patients receive a consultation with our radiation oncologists and then receive radiation therapy close to the patient's home. Therefore, it is possible that differences in radiation therapy could introduce heterogeneity into the histopathologic responses. In our institution, the decision to treat patients with or without neoadjuvant radiation therapy is made by a multidisciplinary team of medical oncologists, radiation oncologists, pathologists, and orthopedic oncologists and is dependent upon various clinical factors. In terms of differences between the two cohorts in this study, patients with NRT were older which could explain the difference in the median overall survival between the two groups, but the difference in OS was not statistically significant (Table 1). While the median follow‐up time between the two groups was different at 3 years for the NRT group and 1.3 years for patients treated without NRT, there was no statistical difference between the follow‐up time based on Student's *t* test analysis. This could be a potential source of bias that may confound trends in recurrence‐free survival. Future prospective studies examining these trends should standardize oncologic follow‐up time in both cohorts to reduce this bias. The univariate analysis of patient demographic factors, such as age, gender, race, and tumor site, did not demonstrate a statistically significant correlation with overall survival or recurrence‐free survival, indicating that they are unlikely confounding variables for this analysis. In addition, tumor subtype and tumor margins did not demonstrate a significant correlation with oncologic outcomes; though this may be due to the low prevalence of certain tumor subtypes within our cohort and the low prevalence of tumor resection with positive margins. Nevertheless, we note that African‐American patients showed a trend for worse oncologic outcomes (Table 2). Racial disparities in oncologic outcomes after treatment of STS have been documented in large registry studies.^17^ Racial equity in outcomes may be achieved by decreasing barriers to diagnosing soft‐tissue sarcomas in African‐American patients and prompt referral to experienced sarcoma centers for multidisciplinary evaluation and treatment.^18^ Future studies on the histopathologic markers identified through our work, such as fibrosis/ hyalinization and infarction, can be analyzed after different types of NRT to investigate the effect of total radiation dose and daily fraction size (i.e., large radiation doses/day with hypofractionated radiotherapy) on the prognostic value of these pathologic markers. In terms of the biologic relationship between the histologic markers identified within this study, further in vitro studies and immunohistochemical studies are needed to clarify how the biologic processes that occur within the tumor microenvironment and its response to radiation may ultimately present in the pathologic markers we described in this study. A problem that is inherent in studying STS is the heterogeneity of the tumor population and the low prevalence of disease that makes studying individual subtypes challenging. To determine if fibrosis/hyalinization and infarction after NRT are associated with outcomes in less‐common STS subtypes, pooling histopathological, and outcome data from multiple institutions would be needed. With regard to the quantitative analysis of the pathologic markers considered here, this study utilized two pathologists when analyzing the tumor specimens. In the future, including more pathologists in grading histopathologic features, assessing interobserver variability, and developing a fully blinded protocol for pathologic assessment could help address any observer bias that may impact the results.

## 5 CONCLUSION

In this work, we have identified pathologic factors such as fibrosis/hyalinization, tumor infarction, and percent of the viable tumor as pathologic characteristics unique to STS tumors treated with NRT. Of these factors, fibrosis/hyalinization and tumor infarction were independently associated with survival. Specifically, patients with tumors exhibiting >12.5% fibrosis/hyalinization and <27% infarction following NRT are associated with higher overall survival and recurrence‐free survival. Although these specific histopathologic measurements were found to be statistically significant in this study, further prospective pathologic studies must rigorously validate these findings to identify reliable pathologic cutoffs. If validated in other studies, fibrosis/hyalinization and tumor infarction could be used in clinical trials to identify patients at higher risk for disease recurrence and risk‐stratify adjuvant therapy. In addition, further analysis of the biology underlying these specific pathologic markers of treatment effect could provide new insights into sarcoma response to radiotherapy and progression, which has the potential to lead to new therapeutic strategies.

## CONFLICT OF INTEREST

DGK is on the scientific advisory board and owns stock in Lumicell, Inc, which is developing intraoperative imaging technology. He is a co‐founder of X‐RAD Therapeutics, which is developing radiosensitizers. He is also a co‐inventor of an intraoperative imaging device and radiosensitizers. He reports research support from Merck, Bristol Myers Squibb, Varian Medical Systems Inc., and X‐RAD Therapeutics.

## ETHICS APPROVAL

Ethical approval for retrospective chart review of patient data was approved by the institutional IRB committee.

## Supporting information

Supplementary Material

## Data Availability

The data that support the findings of this study are available on request from the corresponding author. The data are not publicly available due to privacy or ethical restrictions.
