# Supplementary material for: Extent of tumor fibrosis/hyalinization and infarction following neoadjuvant radiation therapy is associated with improved survival in patients with soft‐tissue sarcoma
Source: Cancer Med. 2021 Nov 27;11(1):194–206. doi: 10.1002/cam4.4428 (PMC8704179; doi:10.1002/cam4.4428)
Supplement: Supplementary file 1 — Supplementary Material [file CAM4-11-194-s001.docx]

1. Coagulative Necrosis

1. Fibrosis/ hyalinization

1. % Viable Tumor

1. % Infarction
